# Surgeon-specific differences in recurrence rates among patients undergoing burr hole evacuation for chronic subdural hematoma

**DOI:** 10.1016/j.bas.2025.105634

**Published:** 2025-10-13

**Authors:** Aron Alakmeh, Vittorio Stumpo, Stefanos Voglis, Antonio Spinello, Victor Gabriel El-Hajj, Adrian Elmi-Terander, Luca Regli, Carlo Serra, Victor E. Staartjes, Flavio Vasella

**Affiliations:** a Machine Intelligence in Clinical Neuroscience & Microsurgical Neuroanatomy (MICN) Laboratory, Department of Neurosurgery, Clinical Neuroscience Center, University Hospital Zurich, University of Zurich, Zurich, Switzerland; b Department of Clinical Neuroscience, Karolinska Institutet, Stockholm, Sweden; c Örebro University, Örebro, Sweden

**Keywords:** Chronic subdural hematoma, Hygroma, Burr hole, Recurrence, Surgeon differences

## Abstract

**Introduction:**

Chronic subdural hematoma (cSDH) frequently recurs. Numerous studies have investigated the influence of various factors on the likelihood of recurrence, yet the potential influence of individual surgeon identity beyond general experience level remains unclear.

**Research question:**

To evaluate whether surgeon-specific differences contribute to recurrence rates whilst accounting for standardized technique and known patient-/procedure-related factors.

**Materials and methods:**

Retrospective analysis of burr hole evacuation for cSDH or hygroma at a single tertiary center. Standardized surgical technique involved two burr holes with subdural lavage and drainage placement. Primary outcome was symptomatic recurrence necessitating redo surgery. Surgeon-specific variability in recurrence was assessed via three statistical methods: risk-standardized observed-to-expected (O/E) ratios, logistic generalized estimating equations (GEE), and hierarchical Bayesian logistic modeling, adjusted for covariates.

**Results:**

Among 116 patients (age = 78.0 years, 75.0 % = male, 26.7 % = bilateral procedures), symptomatic recurrence occurred in 15 cases (12.9 %). Risk-standardized-O/E recurrence ratios varied from 0.00 to 1.65, with wide confidence intervals indicating uncertainty, but all within expected ranges (median O/E = 1.11, IQR = 0.60–1.45). GEE analysis demonstrated significant surgeon-specific clustering (ICC = 0.61, large effect), indicating between-surgeon differences could explain more than half of the remaining variance in recurrence. Bayesian hierarchical modeling showed moderate surgeon-specific clustering with an ICC = 0.14, indicating that between-surgeon differences accounted for approximately 14 % of total variance in recurrence.

**Discussion and conclusions:**

Our study demonstrates modest yet measurable surgeon-specific differences in recurrence rates following standardized burr-hole evacuation for cSDH. These findings support further investigation into surgeon-specific variability, particularly for more complex procedures, to identify actionable technical differences and optimize surgical outcomes universally.

## Highlights

• Our analytical approach demonstrates moderate between-surgeon differences in recurrence rates of chronic subdural hematoma.
• Despite small sample size, findings indicate individual neurosurgical technique influences cSDH outcomes.
• The study lays the groundwork for larger multicenter studies to quantify between-surgeon differences.

## 1 Introduction

Chronic subdural hematoma (cSDH) is one of the most common neurosurgical conditions, with an overall reported incidence of 1–10/100,000 per year (Yadav et al., 2016; Adhiyaman et al., 2017). In individuals over the age of 65, the reported incidence rises substantially, reaching up to 58.1/100,000 per year (Stubbs et al., 2021). Driven by the aging population, the incidence of cSDH is projected to increase by 50 % over the next two decades (Stubbs et al., 2021).

The current treatment of choice for uncomplicated cSDH is burr hole evacuation (Yadav et al., 2016). However, a major postoperative complication remains the recurrence of cSDH with reported rates ranging from 5 to 20 % across studies (Omerhodžić et al., 2024; Sun et al., 2005; Chan et al., 2015). Numerous studies have suggested and analyzed potential risk factors for recurrent cSDH after burr hole evacuation (Edlmann et al., 2019; Hounkpatin et al., 2024; Hammer et al., 2017; Staartjes et al., 2024). While factors such as bilaterality, surgical techniques, different pre- and postoperatively assessed clinical scores and recovery protocols have been investigated extensively, the literature investigating the association between cSDH recurrence rates and the surgeon's experience level as well as experience-independent surgeon-specific differences remains scarce.

To date, only five studies have investigated the association between cSDH recurrence rates and the surgeon's experience level, with just three directly comparing cSDH recurrence rates between surgeries performed by inexperienced junior versus experienced senior surgeons (Mellergård and Wisten, 1996; Gastone et al., 2004; Phang et al., 2015; Maldaner et al., 2018; Adegboyega et al., 2024). The first comparative study on surgical experience in cSDH recurrence rates was done by Mellergård et al., in 1996 and surprisingly showed significantly lower recurrence rates in non-experienced surgeons compared to experienced senior surgeons (Mellergård and Wisten, 1996). The authors explained this seemingly paradox finding as a reflection of evolving techniques adopted by junior staff over the 25-year study period. This observation may also, in part, be explained by the fact that patients treated by senior surgeons perhaps present with higher surgical or medical complexity. Subsequent studies by Gastone et al. and Phang et al. found no significant differences in recurrence rates; however, it is noteworthy that only Phang et al. directly compared two surgeon groups, while Gastone et al. analyzed recurrence rates in inexperienced surgeons relative to global averages (Gastone et al., 2004; Phang et al., 2015). The most recent study on this topic by Maldaner et al. similarly showed no difference in revision surgery rates between supervised resident surgeons and board-certified surgeons (Maldaner et al., 2018). Overall, the limited available evidence suggests that there is no higher cSDH recurrence risk in surgeries performed by inexperienced surgeons. Consequently, burr hole evacuation of cSDH is often among the first cranial procedures taught during neurosurgical training.

However, no study has investigated the association between cSDH recurrence rates and the experience-independent surgeon-specific variability to date. Thus, while existing studies suggest that surgeon experience level does not significantly impact recurrence rates, it remains unknown whether surgeon-specific variability exists when accounting for surgeon experience, patient baseline characteristics and following standard procedure. This study therefore aims to fill this gap of knowledge by analyzing surgeon-specific variability in recurrence rates among patients operated for cSDH whilst accounting for different experience levels, patient baseline characteristics and following standard procedure.

## 2 Materials and Methods

### 2.1 Overview and data collection

To evaluate real-world procedural data, we analyze a consecutive cohort of all adult patients who have undergone burr hole surgery for subdural chronic hematoma or hygroma at the Department of Neurosurgery, University Hospital Zurich, from October 2021 up to June 2023. We included only patients with a complete six-week follow-up visit. We excluded procedures done for subdural empyema/abscesses or those primarily planned as craniotomies. Data extraction is based on our prospective institutional patient registry, which collects demographic, procedural, adverse events, and clinical outcome data. Imaging results and data on anticoagulants/antiaggregants are retrospectively added, as well as missing data wherever feasible. The scientific workup is approved by the local ethics review board (Kantonale Ethikkommission Zürich PB-2017-00093) and registered internationally at clinicaltrials.gov (NCT01628406). This report is compiled according to the STROBE (Strengthening the Reporting of Observational Studies in Epidemiology) statement (Von Elm et al., 2007).

### 2.2 Surgical technique

It is to be noted that surgical technique was standardized among all operators. The patient is positioned in supine or semilateral position with the upper body and head slightly elevated and turned so that the frontal burr hole corresponds to the highest point of the head. For bilateral procedures, the patient is positioned supine with the head on a half-moon headrest. The Stephanion and parietal tuberosity are identified, and short skin incisions made over each. After dissecting the periosteum, 14-mm burr holes are placed over the Stephanion and the parietal tuberosity. Hemostasis is achieved using bone wax. The dura is incised in a cruciform fashion and coagulated, first frontally and then parietally. Ringer's solution at body temperature is flushed from both burr holes until good communication is achieved between the two and until the solution flows back clearly. A Redon drainage is then placed subperiosteally, covering both burr holes. The parietal incision is then closed with inverted galeal sutures and the skin closed with staples. Finally, the remaining subdural space is filled with Ringer's solution until no air remains subdurally, and the frontal incision is closed in the same fashion. Postoperative management was carried out according to our previously published protocol (Staartjes et al., 2024).

### 2.3 Outcome measures

#### 2.3.1 Baseline characteristics

Apart from basic demographic characteristics, we collected unilateral or bilateral surgery, prior surgery for cSDH, surgical time in minutes, and preoperative presence of anticoagulants such as Vitamin K inhibitors or direct FXa inhibitors, as well as antiaggregants such as acetylsalicylic acid or ADP/P2Y inhibitors. Other predictors included admission Glasgow Coma Scale (GCS) and surgeon experience level, which was coded in three ordinal levels (1 - junior residents with <2 years of experience, 2 - senior residents with >2 years of experience, and 3 - board-certified neurosurgeons).

#### 2.3.2 Primary endpoint

The primary endpoint was symptomatic recurrence of the cSDH requiring re-evacuation. While imaging-based outcomes were available, we chose this “hard” outcome due to its clinical importance and secure collection, as our center is the only neurosurgical center within our catchment area and all patients operated for cSDH are seen at follow-up, and any recurrent symptomatic patients would be cared for by our center.

### 2.4 Statistical analysis

Categorical variables are reported as numbers (percentages). Continuous data are reported as means and standard deviations or medians and interquartile ranges. Analyses are carried out per procedure – not per patient. Complete case analysis was performed, no imputation was carried out.

To elucidate between-surgeon variation in recurrence rates, we relied upon three different methods.

#### 2.4.1 Risk-standardized observed/expected ratios

First, we calculated risk-standardized observed and expected (O/E) event ratios by fitting an ordinary logistic regression model without surgeon term, with covariates (confounders) potentially relevant to recurrence including presence of anticoagulant or antiaggregant drugs, age, bilateral surgery, prior cSDH surgery, admission GCS, and duration of the surgery. Here, we also included the ordinal level of experience, as we wanted to separate between-surgeon variation by taking away measurable confounders such as level of experience. We then calculated 95 % confidence intervals (CIs) around each O/E ratio using log-normal approximation and used exact one-sided Poisson upper limits for surgeons without any observed recurrences. Finally, we plotted the expected versus observed ratios per surgeon in a funnel-type plot.

#### 2.4.2 Generalized estimating equations to estimate generalized clustering

Second, we applied methods to estimate marginal clustering, in this case logistic generalized estimating equations (GEEs) with exchangeable working correlation structures. This enables the estimation of regression coefficients for all covariates (confounders as described above) plus a single working correlation parameter, namely α^. Because the α^ of an exchangeable GEE equals the average intraclass correlation coefficient (ICC) between different patients of the same surgeon, it effectively displays correlation of patients within a single surgeon (“within-cluster”). A large within-cluster (within-surgeon) correlation that is independent of/unexplained by covariate effects thus means a relevant between-surgeon difference. Hence, when α^ is large, it is because between‐surgeon differences explain most of the residual variability (marginal clustering). The resulting α^ = ICC can then be interpreted like other Pearson-style correlations (<0.10 negligible; 0.10–0.30 small; 0.30–0.50 moderate; ≥0.50 large) according to the framework set by Cohen for true correlation markers (Cohen, 1992).

#### 2.4.3 Hierarchical Bayesian logistic models for surgeon-specific clustering

Third, to enable not only interpretation of marginal clustering (generalized between-surgeon effect) but to model every surgeon, we also used a Bayesian approach for subject-specific (surgeon-specific) clustering. Here, we fit a Bayesian hierarchical (multi-level) logistic model to assess surgeon-specific residual risk for recurrence. Concretely, we model log-odds of recurrence for each patient as a combination of their covariates (confounders, as described above) plus a surgeon-specific intercept. The result is a full posterior distribution for *σ*_*u*_, which is the standard deviation of the surgeon intercepts on the log‐odds scale. Thus, *σ*_*u*_ explains how many log-odds units surgeons differ, with larger values signifying that surgeons differ more. *σ*_*u*_ can then be converted to a latent-scale ICC by expressing it as a proportion of the total variance. The resulting ICC equals the fraction of the overall variability in patient-level log-odds of recurrence that can be attributed to *between*-surgeon differences. True ICCs in multi-level models are not interpreted using correlation-style rules, but instead commonly using the standards set by Koo and Li (<0.50 poor; 0.50–0.75 moderate; 0.75–0.90 good; ≥0.90 excellent) (Koo and Li, 2016).

All analyses were performed using version 4.4.2 of R (R Foundation for Statistical Computing, Vienna, Austria; https://www.R-project.org/) (R Core Team, 2024). A 2-tailed *P* ≤ 0.05 was considered statistically significant.

## 3 Results

**Fig. 1:**
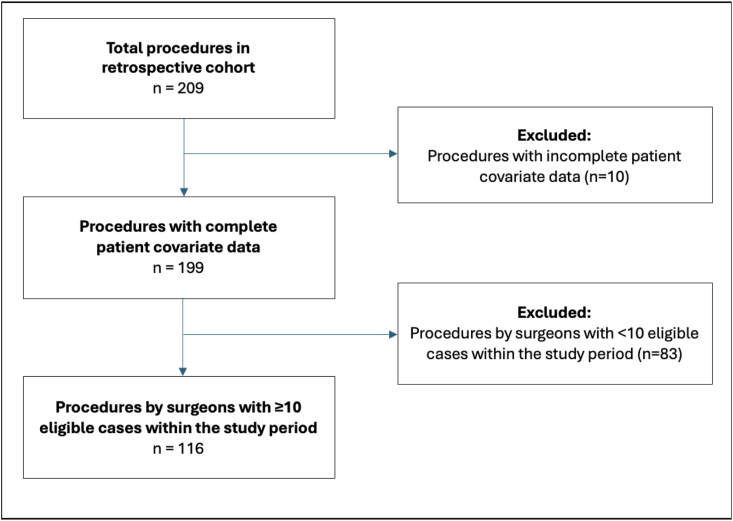
Flowchart demonstrating the flow of procedures collected in the retrospective cohort up to statistical analysis.

Patient flow is demonstrated in Fig. 1. A total of 116 patients had complete covariate data and were treated by a surgeon who had performed at least 10 eligible surgeries within the data collection timeframe. Detailed baseline data is provided in Table 1. The median age was 78.0 years (IQR 69.0–84.0). A total of 87 (75.0 %) patients were male, and 31 (26.7 %) underwent bilateral evacuation. Median operative duration was 52.0 min (IQR 40.0–64.0), and the mean admission GCS was 13.9 ± 2.5. Prior cSDH surgery had been performed in 12 (10.3 %) patients. In terms of coagulation-active medications, 30 (25.9 %) patients were on systemic anticoagulation and 25 (21.6 %) on antiplatelet therapy. Overall, 15 (12.9 %) patients experienced recurrence in the form of a symptomatic recurrent subdural hematoma requiring redo surgery.

**Table 1:** Summary of baseline characteristics.

Parameter	Total (N = 116)
Patients of male sex, n (%)	87 (75.0)
Patients with prior cSDH, n (%)	12 (10.3)
Patients with bilateral cSDH surgery, n (%)	31 (26.7)
Patients on anticoagulation therapy, n (%)	30 (25.9)
Patients on antiplatelet therapy, n (%)	25 (21.6)
cSDH recurrence, n (%)	15 (12.9)
Surgery duration [min], (median [IQR])	52.00 [40.00, 64.00]
Patient age [y], (median [IQR])	78.00 [69.00, 84.00]
GCS at admission, mean (SD)	13.89 (2.45)
Surgeon experience levels, n (%)	
Junior resident	42 (36.2)
Senior resident	50 (43.1)
Board-certified surgeon	24 (20.7)
Patients operated by surgeon, n (%)	
Surgeon number 1	14 (12.1)
Surgeon number 2	22 (19.0)
Surgeon number 3	13 (11.2)
Surgeon number 4	14 (12.1)
Surgeon number 5	12 (10.3)
Surgeon number 6	10 (8.6)
Surgeon number 7	19 (16.4)
Surgeon number 8	12 (10.3)

cSDH, chronic subdural hematoma; IQR, Interquartile range; SD, standard deviation; GCS, Glasgow coma scale.

Eight surgeons qualified for the study, including 3 junior and 3 senior residents as well as 2 board-certified neurosurgeons. Surgeon experience levels were distributed as follows: 42 (36.2 %) procedures performed by junior residents, 50 (43.1 %) by senior residents, and 24 (20.7 %) by board-certified neurosurgeons. Patient volumes per surgeon ranged from 10 to 22 performed evacuations.

### 3.1 Risk-standardized observed/expected ratios

**Fig. 2:**
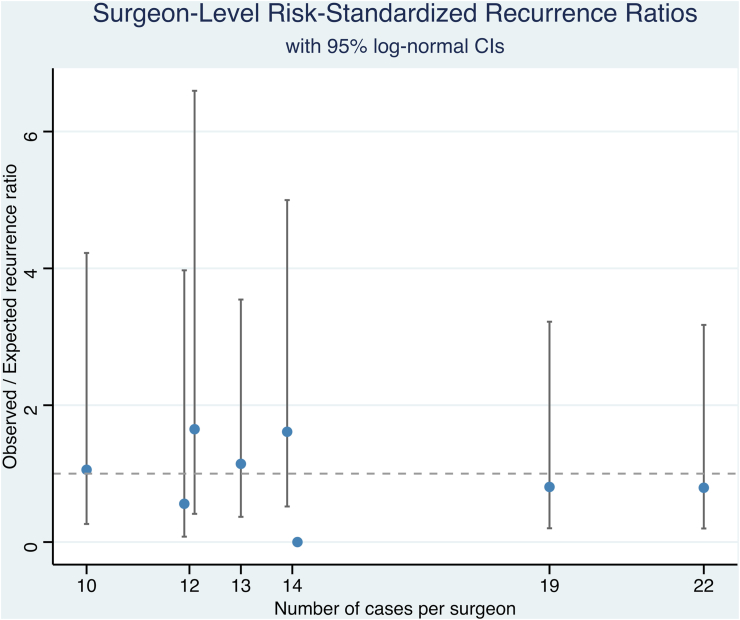
Surgeon-level risk-standardized recurrence ratios (observed versus expected recurrences) provided with 95 % log-normal confidence intervals.

A table of these results is provided in Table 2 and visualized in Fig. 2. Here, we saw minor differences among surgeons in terms of observed versus expected recurrence (O/E) ratios. O/E ratios ranged from 0.00 to 1.65, with wide confidence intervals (CIs) reflecting small event counts and large uncertainty. None of the surgeons’ CIs excluded 1, meaning that all estimated O/E ratios were within the expected range. The median O/E recurrence ratio across the eight surgeons was 1.11 (IQR: 0.60–1.45), reflecting a well-calibrated risk standardization model.

**Table 2:** Comparison of observed and expected (modeled) recurrence rates among surgeons.

Surgeon	Cases operated	Observed Recurrences	Expected Recurrences	O/E Ratio	Logarithmic SE	LCL	UCL
Surgeon number 1	14	3	1.48	2.02	0.58	0.65	6.26
Surgeon number 2	22	2	2.23	0.90	0.71	0.23	3.59
Surgeon number 3	13	3	2.20	1.36	0.58	0.44	4.23
Surgeon number 4	14	0	1.29	0.00	0.00	0.00	4.84^a^
Surgeon number 5	12	1	1.69	0.59	1.00	0.08	4.19
Surgeon number 6	10	2	1.51	1.33	0.71	0.33	5.31
Surgeon number 7	19	2	3.30	0.61	0.71	0.15	2.43
Surgeon number 8	12	2	1.31	1.53	0.71	0.38	6.12

SE, standard error; O/E, Observed/Expected; LCL, lower confidence limit; UCL, upper confidence limit.

a Exact Poisson upper limit.

### 3.2 Generalized clustering

The logistic GEEs working correlation estimate was an α^ of 0.61 with a standard error (SE) of 0.143, which by definition equals the ICC among patients of the same surgeon. An ICC of 0.61 corresponds to large clustering (ICC ≥0.50), indicating that over 60 % of residual variation in recurrence is shared within surgeons. Looking at the fitted estimates for the covariates, bilateral surgery was significantly protective of recurrence (Odds ratio: 0.76; 95 % CI 0.62–0.93; p = 0.008), and each one‐point increase in admission GCS was associated with a 6 % lower odds of recurrence (Odds ratio: 0.94 per point; 95 % CI 0.90–0.99; p = 0.012). These results are also demonstrated in Table 3.

**Table 3:** Association between covariates and cSDH recurrence (GEE and Bayesian Analysis).

Covariates	GEE OR (95 % CI)	*p*	Bayes posterior median OR (95 % CrI)
GCS at admission	0.94 (0.90–0.99)	0.012∗	0.67 (0.48–0.89)
Bilateral surgery	0.76 (0.62–0.93)	0.008∗	0.02 (0.00–0.48)
Patient on anticoagulation	0.95 (0.80–1.13)	0.550	0.45 (0.07–2.60)
Patient on antiaggregation	0.87 (0.75–1.01)	0.063	0.22 (0.03–1.80)
Patient age [y]	0.99 (0.98–1.00)	0.062	1.00 (0.99–1.00)
Prior cSDH surgery	1.02 (0.69–1.51)	0.928	1.82 (0.30–11.20)
Surgery duration [min]	1.00 (0.999–1.009)	0.078	1.00 (1.00–1.01)
Surgeon experience level			
Senior vs junior resident	0.63 (0.08–4.75)	0.652	0.45 (0.09–2.30)
Board-certified surgeon vs junior resident	1.01 (0.12–8.53)	0.995	2.01 (0.25–14.50)

cSDH, chronic subdural hematoma; GEE, Generalized Estimating Equations; OR, Odds ratio; CI, confidence interval; CrI, credible interval, GCS, Glasgow coma scale.

∗*p* ≤ 0.05.

### 3.3 Surgeon-specific clustering

The Bayesian logistic mixed model with surgeon random intercept produced a posterior median standard deviation of the intercepts *σ*_*u*_ of 0.72. This corresponds to a latent-scale ICC of 0.14, indicating a moderate between-surgeon effect. Consequently, roughly 14 % of total latent variance in recurrence risk is attributable to between-surgeon differences. The 95 % credible intervals in Table 3 demonstrate, just as in the GEE approach, that bilateral surgery and higher GCS were associated with decreased likelihood of recurrence.

## 4 Discussion

This study is the first to demonstrate measurable differences in recurrence rates between individual surgeons performing burr-hole evacuation of cSDH. Prior investigations primarily focused on surgeon experience level or institutional factors, without examining surgeon-specific variability directly (Mellergård and Wisten, 1996; Gastone et al., 2004; Phang et al., 2015; Maldaner et al., 2018). Notably, a recent meta-analysis reported no significant difference in recurrence rates between senior attending and junior resident neurosurgeons, suggesting experience alone does not fully explain recurrence outcomes (Adegboyega et al., 2024). Consistent with their findings, our analysis likewise showed no significant differences in recurrence rates across surgeon experience levels, which is partly attributable to the highly standardized and relatively non-complex nature of the procedure. Our findings however extend beyond these previous analyses, showing a modest surgeon-specific effect on recurrence risk even when accounting for surgeon experience and with surgeons following a standardized technique. Thus, individual surgeon identity appears to influence outcomes independently of training level.

The magnitude of the surgeon-specific effect we observed was small yet clearly measurable with substantial variability in recurrence outcomes across surgeons' risk-standardized observed-to-expected (O/E) recurrence ratios ranging from 0.00 to 1.65 (median 1.11). These findings indicate that while some surgeons achieved recurrence rates below expected benchmarks, others exceeded them. As expected, most of the observed difference in outcome between surgeons can be attributed to patient- and disease-specific factors. For example, higher admission GCS scores correlated with lower recurrence risk (OR 0.94; 95 % CI 0.90–0.99; p = 0.012). Interestingly, although patients with bilateral cSDH would be expected to show similar or even higher recurrence rates than in unilateral cases, our finding of a significantly reduced recurrence risk (OR 0.76; 95 % CI 0.62–0.93; *p* = 0.008) lacks a clear explanation and should therefore be interpreted with caution (Miah et al., 2021). It is also noteworthy that the implementation of the new enhanced recovery protocol after surgery (ERAS) did not, consistent with previous studies, have any measurable impact on recurrence rates (Staartjes et al., 2024). Of the included patients, 52 were treated before and 64 after the introduction of ERAS at our center.

The magnitude of surgeon-specific variability was quantitatively confirmed through two complementary analytical methods: generalized estimating equations (GEE) and Bayesian hierarchical modeling. GEE analysis revealed a large surgeon-specific clustering effect (intraclass correlation coefficient [ICC] 0.61), indicating that approximately 61 % of residual variance in recurrence rates is attributable to differences between individual surgeons. Bayesian modeling yielded a more moderate ICC of 0.14, suggesting methodological differences between population-averaged (GEE) and surgeon-specific (Bayesian) models. Nonetheless, both analyses consistently demonstrated a significant, though modest, surgeon-specific influence on recurrence outcomes. Notably, no surgeon emerged as a statistical outlier with significantly divergent recurrence rates after adjustment for patient and procedural factors. Rather, variability was subtle and distributed across all surgeons. This absence of extremes suggests the surgeon effect is not driven by exceptional cases but represents a broader phenomenon of minor differences among competent surgeons. Practically, this indicates that while no surgeon performed notably poorly, subtle differences in practice could cumulatively influence outcomes. Consequently, this underlines potential benefits from further refining and standardizing surgical practices, even when overall competence is high.

Several potential explanations may account for these surgeon-specific differences, despite the standardized surgical protocol at our institution, including variations in surgical technique or postoperative care practices not covered by high-quality evidence. Numerous technical aspects of cSDH evacuation have been specifically investigated resulting in recommendations. Among them, for example, randomized clinical trials provided evidence to support both thorough intraoperative irrigation (Raj et al., 2024) as well as the placement of postoperative drains, as these were associated with a significant decrease in cSDH recurrence rates (Soleman et al., 2019). Similarly, another study suggested that subperiosteal drains may offer comparable recurrence reduction to traditional subdural drains while reducing associated complications such as infection or cortical injury (Soleman et al., 2019).

Beyond intraoperative and technical aspects, postoperative management protocols including patient positioning, timing of mobilization, and anticoagulant resumption, have been shown to influence outcomes. For instance, early postoperative mobilization has not been associated with increased recurrence and significantly reduces medical complications such as pneumonia and venous thromboembolism (Sousa et al., 2023). Although routine postoperative imaging is not performed at our institution due to probable lack of clinical benefit (Schucht et al., 2019), variability in surgeons' threshold for postoperative imaging and subsequent reoperation decisions could still introduce outcome differences. It is evident that even highly standardized procedures such as cSDH evacuation can differ subtly in practice, such as variations in the thoroughness of subdural cavity irrigation, incision of hematoma membranes, or meticulousness of subcutaneous hemostasis. Addressing these procedural nuances through enhanced standardization might further minimize recurrence variability and improve patient outcome.

Our findings have broader implications, as the existence of surgeon-specific differences in a relatively straightforward neurosurgical procedure such as cSDH evacuation suggests potentially greater variability in more technically complex surgeries. Burr-hole drainage for cSDH is often considered a basic operation, frequently delegated to relatively junior surgeons in training (Maldaner et al., 2018; Adegboyega et al., 2024). While it might be presumed that a simple, standardized operation leaves minimal room for individual surgeon influence, our data imply otherwise. It is probable that there are even greater inter-surgeon outcome differences in technically more demanding procedures, such as aneurysm clipping, complex spine surgery, or resections of eloquent tumors. Indeed, substantial evidence from surgical literature confirms that objectively assessed technical skills significantly impact patient outcomes. For example, blinded expert review of operative technique correlates strongly with lower complication and reoperation rates (Stulberg et al., 2020). Additionally, surgeons who focus their practice within specialized subspecialties consistently demonstrate better clinical outcomes, lower morbidity, and mortality rates compared to generalists, independent of case volume alone (McCutcheon et al., 2018; Sahni et al., 2016). Within this context, further investigation of surgeon-specific differences may lead to the identification of specific underlying practices in surgical technique or postoperative management associated with better patient outcomes, thus enabling the implementation of such practices either routinely or within tailored surgeon-specific training programs to ultimately enhance patient outcome.

Overall, this paper was aiming to analyze if there even are quantifiable differences between surgeons independent of experience level and patient risk factors. Future studies are needed to analyze why these differences occur while possibly investigating other factors such as the influence of surgical assistants and anesthesiologists.

## 5 Limitations

Several limitations must be considered in the context of the present study. First, our definition of recurrence required repeat surgical intervention, potentially differing from other studies using radiographic or clinically conservative definitions. Thus, direct comparisons with studies employing alternative definitions should be made cautiously. Additionally, despite standardizing the surgical approach, detailed procedural nuances such as precise irrigation methods, drain placement specifics, or subtle postoperative care variations were not captured systematically. These unmeasured differences may explain part of the observed surgeon-specific variability. Furthermore, as a single-center study, generalizability is limited, since practices and patient demographics may differ elsewhere. We also acknowledge that volumetric data of the cSDH would have provided additional value for the analysis.

Surgical experience was only approximated and categorized by years in practice, as is commonly done in previous studies. However, this measure does not fully capture surgical expertise, since actual surgical experience and procedural volume may vary considerably between surgeons in the same training phase. Moreover, information on the extent of supervision or partial assistance by senior surgeons, particularly in the case of junior residents, was not available in our dataset.

Specific details regarding surgical technique, such as thoroughness of subdural cavity, irrigation, incision of hematoma membranes, or meticulousness of subcutaneous hemostasis were not investigated in this study. Given that inter-surgeon differences were observed in this study, precisely these subtle technical aspects could be addressed in future investigations to further optimize and standardize this already highly standardized procedure – and to arrive at potential explanations for our findings. Further factors, such as inter-individual differences among anesthesiologists and surgical assistants, were not assessed and may also be addressed in future investigations.

Methodologically, differences in ICC estimates between the GEE and Bayesian approaches highlight the sensitivity of variance quantification to analytical methods. While primary outcome data were prospectively collected, supplementary data such as perioperative variables were partially collected retrospectively, potentially introducing minor inaccuracies or data gaps. Lastly, our moderate sample size limits statistical power, potentially obscuring smaller effects or nuanced differences between surgeons. Larger cohorts would enhance statistical power and allow for more granular analysis.

## 6 Conclusions

In conclusion, our study identified modest yet significant surgeon-specific differences in symptomatic recurrence following standardized burr hole evacuation for cSDH. These findings highlight the importance of acknowledging and addressing surgeon-specific factors through targeted quality improvement and educational initiatives. Further investigation into surgeon-specific variability, particularly for more complex procedures, is warranted to identify relevant differences and optimize surgical outcomes universally.

## Grants and support

This research did not receive any specific grant from funding agencies in the public, commercial, or not-for-profit sectors.

## Conflict of interest

The authors declare that the article and its content were composed in the absence of any commercial or financial relationships that could be construed as a potential conflict of interest.
